# Subcellular Localization of Total and Activated Src Kinase in African American and Caucasian Breast Cancer

**DOI:** 10.1371/journal.pone.0033017

**Published:** 2012-03-22

**Authors:** Muralidharan Anbalagan, Krzysztof Moroz, Alaa Ali, Latonya Carrier, Seth Glodowski, Brian G. Rowan

**Affiliations:** 1 Department of Structural and Cellular Biology, Tulane University School of Medicine, New Orleans, Louisiana, United States of America; 2 Section of Surgical Pathology and Cytopathology, Tulane University School of Medicine, New Orleans, Louisiana, United States of America; University of Texas Health Science Center at Houston, United States of America

## Abstract

**Background:**

Src, a non-receptor tyrosine kinase is elevated in cancer with expression and activity correlated with cell proliferation, adhesion, survival, motility, metastasis and angiogenesis. There is limited data on Src expression and subcellular localization in breast cancer and no information about expression in racial/ethnic groups.

**Methodology/Principal Findings:**

The present study evaluated Src expression, activity, and subcellular localization in triple negative breast cancer (TNBC) and ERα positive breast cancer (ER+BC), cancer tissue and adjacent normal epithelial ducts, and Caucasian and African American cases. 79 paraffin embedded breast carcinoma cases were obtained from Tulane University Hospital between 2007–2009. 39 cases represented TNBC (33-African Americans, 4-Caucasians, 2-unknowns) and 40 cases represented ER+BC (21-African Americans, 16-Caucasians, 3-unknowns). Immunohistochemistry was used to measure staining distribution and intensity of total Src and activated phospho-SrcY416 (p-Y416Src) in carcinoma tissue and adjacent normal mammary ducts. In TNBC and ER+BC, total Src was significantly higher in cancer compared to adjacent normal ducts (P<0.0001) in both cell membrane and cytoplasm. In membranes, p-Y416Src was elevated in cancer compared to normal ducts. Total Src in the tumor cytoplasm was significantly higher in TNBC compared to ER+BC (P = 0.0028); conversely, p-Y416Src in the tumor cell membranes was higher in TNBC compared to ER+BC (P = 0.0106). Comparison between African American (n = 21) and Caucasian ER+BC (n = 16) revealed no significant difference in expression and localization of total Src and p-Y416Src. TNBC cases positive for lymph node metastasis showed elevated membrane p-Y416Src compared to lymph node negative TNBC (P = 0.027).

**Conclusion/Significance:**

Total Src and p-Y416Src were expressed higher in cancer compared to adjacent normal ducts. Cytoplasmic total Src and membrane p-Y416Src were significantly higher in TNBC compared to ER+BC. TNBC cases with lymph node metastasis showed elevated membrane p-Y416Src. Taken together, Src was elevated in the membrane and cytoplasm of more aggressive TNBC.

## Introduction

Triple negative breast cancer (TNBC) are tumors that lack expression of estrogen receptor α (ERα), progesterone receptor (PR), and amplification/overexpression of human epidermal growth factor receptor 2 (HER2/neu). TNBC comprises 15% of all breast cancers and occurs with greater frequency in younger patients and in African American women [1], [2]. TNBC exhibits a more aggressive phenotype and patients have poor prognosis and shorter time to recurrence compared to ERα positive breast cancer (ER+BC) [3], [4]. Only 30% of women with metastatic TNBC survive 5 years, and many patients eventually die of their disease [4]. Patients with TNBC have been difficult to treat due to heterogeneity within the tumors and lack of definitive targets for development of targeted therapeutics [5], [6]. This lack of effective targeted therapies for TNBC, the disproportionate incidence of TNBC in African American women, and the earlier age of onset highlights an urgent need for novel therapeutic strategies for TNBC [7].

c-Src is an oncogenic non-receptor tyrosine kinase that is up-regulated in approximately half of all breast cancers [8], [9], [10]. A number of studies have demonstrated an elevation in the level and/or activity of Src kinase during progression of breast cancer and numerous reports and commentary highlight the value of targeting Src in breast cancer [11], [12], [13]. Subcellular localization of Src is a key regulatory mechanism for control of Src activation. Src remains inactive in the cytoplasm when it is phosphorylated at Y530; once dephosphorylated, Src translocates to the cell membrane and becomes fully activated by autophosphorylation at Y416 [14], [15], [16], [17]. The activated Src (p-Y416Src) at the cell membrane initiates intracellular signal transduction pathways that influence cell proliferation and adhesion that contributes to cell growth and migration [18].

Src has been associated with breast cancer proliferation, motility and migration/invasion [19], [20], [21]. The role of Src in proliferation, migration and invasion coupled with the elevated Src expression in breast cancer combine to make inhibition of Src a promising target for development of therapeutics. Most importantly, inhibition of Src has recently been identified as a therapeutic target for basal breast cancers including the TNBC subtype [22], [23]. It will be critical to identify breast cancer patients who may benefit from therapy directed at inhibition of Src kinase.

There are few published studies on the expression of Src kinase in clinical breast cancer [24], [25], [26], [27]. No studies have compared TNBC and ER+BC for subcellular localization of Src, Src expression in tumor versus normal cells, or racial/ethnic differences in Src expression. Elevated Src expression and activity, and subcellular localization of Src to the membrane may identify a subset of breast cancer patients who will respond favorably to Src targeted therapy. The present study was undertaken to systematically compare expression, activity and subcellular localization of Src in TNBC and ERα positive breast cancers.

## Results

### Src expression in breast cancer tissue and adjacent normal ducts in ER+BC and TNBC

**Figure 1:**
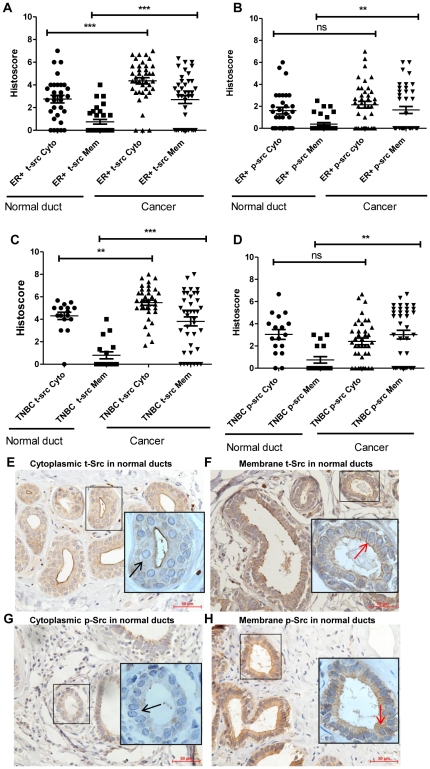
Src expression in breast cancer tissue and adjacent normal ducts of ER+BC and TNBC. A) Comparison between total Src (t-Src) subcellular localization in cytoplasm (Cyto) and membrane (Mem) of normal ducts and cancer of ER+BC; B) Comparison between p-Y416Src (p-Src) expression in cytoplasm and membrane of normal ducts and cancer of ER+BC; C) Comparison between total Src expression in cytoplasm and membrane of normal ducts and cancer of TNBC; D) Comparison between p-Src expression in cytoplasm and membrane of normal ducts and cancer in TNBC. Statistical difference in the distribution of total and p-Y416Src kinase in normal ducts and cancer was analyzed by the Mann-Whitney *U* test, * P<0.05, **P<0.01, *** P<0.001. All error bars represent the SEM (standard error of mean). E) A representative photomicrograph from a TNBC case showing a weak cytoplasmic (1+) t-Src staining in normal ducts as indicated by a black arrow. F) A representative photomicrograph from a TNBC case showing a weak membrane (1+) t-Src staining in normal ducts as indicated by a red arrow (original magnification ×200). G) A representative photomicrograph from a TNBC case showing a weak cytoplasmic p-Src staining (1+) in normal ducts as indicated by a black arrow. F) A representative photomicrograph from a TNBC case showing a weak membrane (1+) p-Src staining in normal ducts as indicated by a red arrow (original magnification ×200) *Scale bar*, *50 *µ*m*. Insets are magnified images from selected areas *(small squares)* to show the membrane and cytoplasmic Src staining clearly.

Table 1 summarizes the clinical-pathological features of the breast cancer cases used in this study. Src expression in cancer cells and in adjacent normal epithelial ducts was scored and compared in tissue sections from resected tumors of BC patient seen in the Tulane University Hospital and Clinics, New Orleans, LA. Sections were stained with an antibody directed against total Src protein, and with an antibody that interacted with Src phosphorylated at tyrosine (Y) 416 which represents the active form of Src kinase. In ER+BC cases, statistical analysis revealed that total Src expression was significantly higher in cancer tissue when compared to adjacent normal epithelial ducts in both the cell membrane (P<0.0001) and the cell cytoplasm (P<0.0001) (Fig. 1A). Phospho-Src (p-Y416Src) was more intensely stained in the cell membrane of cancerous tissue compared to normal ducts (P = 0.0076), whereas there was no difference in the expression of p-Y416Src between cytoplasm of normal ducts and cancer tissue (P = 0.2227) (Fig. 1B). Src was not detected in the cell nucleus for any of the ER+BC or TNBC cases.

**Table 1:** Patient and tumor characteristics.

Patient and tumor characteristics	Variables	TNBC (n = 39)	ER+ BC (n = 40)
**Patient age**	≤50	19	11
	>50	20	29
**Race**	African American	33	21
	Caucasian	4	16
	Others	2	3
**Grade**	Grade 1	1	9
	Grade 2	7	19
	Grade 3	28	7
**Histological type**	Invasive ductal carcinoma	33	29
	Invasive lobular carcinoma	0	4
	DCIS	3	5
	Others	3	2
**Lymph node status**	Negative	10	8
	Positive	13	8
	Not tested	16	24
**Ki67 status**	Low (≤20%)	4	25
	High (>20%)	31	13
	Unknown	4	2

Clinical-pathological characteristics of TNBC and ER+BC cases.

n- number of cases.

Similar to ER+BC, in TNBC cases total Src expression was significantly higher in cancer tissue when compared to adjacent normal ducts in both the cytoplasm (P = 0.0016) and the membrane (P<0.0001) (Fig. 1C). Strong membrane p-Y416Src staining was observed in cancer compared to normal ducts (P = 0.0014); however similar to ER+BC, there was no difference in levels of cytoplasmic p-Y416Src between normal ducts and cancer (P = 0.2502) (Fig. 1D). Figure 1E, F shows representative total Src in the cell cytoplasm and cell membrane respectively of the adjacent normal ducts of TNBC. Figure 1G, H shows representative p-Y416Src in the cytoplasm and membrane respectively of the adjacent normal ducts of TNBC.

### Total Src expression and subcellular localization in cancer cells

95% (37/39) of TNBC cases demonstrated positive total-Src staining in the cytoplasm and 79% (31/39) of TNBC cases demonstrated positive total-Src staining in the membrane (Table 2). 90% (36/40) of ER+BC cases showed cytoplasmic staining for total-Src and 70% (28/40) of ER+BC cases showed membrane staining for total Src (Table 2).

**Table 2:** Subcellular localization of total Src and p-Y416Src in TNBC and ER+BC cases.

Variable	TNBC (n = 39)	ER+ BC (n = 40)	P value
Cytoplasmic total Src	95% (37/39)	90% (36/40)	0.413
Membrane total Src	79% (31/39)	70% (28/40)	0.332
Cytoplasmic p-Y416Src	79% (31/39)	68% (27/40)	0.227
Membrane p-Y416Src	67% (26/39)	40% (16/40)	0.017*

The Chi-square test was used to analyze the frequency of Src distribution and determine differences between TNBC and ER+BC cases.

* P<0.05 was considered to be a statistically significant difference in the distribution of Src between TNBC and ER+BC cases.

### p-Y416Src expression and subcellular localization in cancer cells

79% (31/39) of TNBC cases stained positive for cytoplasmic p-Y416Src and 68% (27/40) of ER+BC cases showed positive staining for cytoplasmic p-Y416Src (Table 2). TNBC cases exhibited membrane p-Y416Src in 67% (26/39) cases whereas only 40% of the ER+BC cases showed membrane p-Y416Src staining (Table 2). The Chi-square test revealed that membrane p-Y416Src was detected more frequently in TNBC cases (67%, 26/39) than in ER+BC (40%, 16/40) (P = 0.0176) (Table 2).

### Src and p-Y416Src expression and subcellular localization in ER+BC among African Americans and Caucasians cases

**Figure 2:**
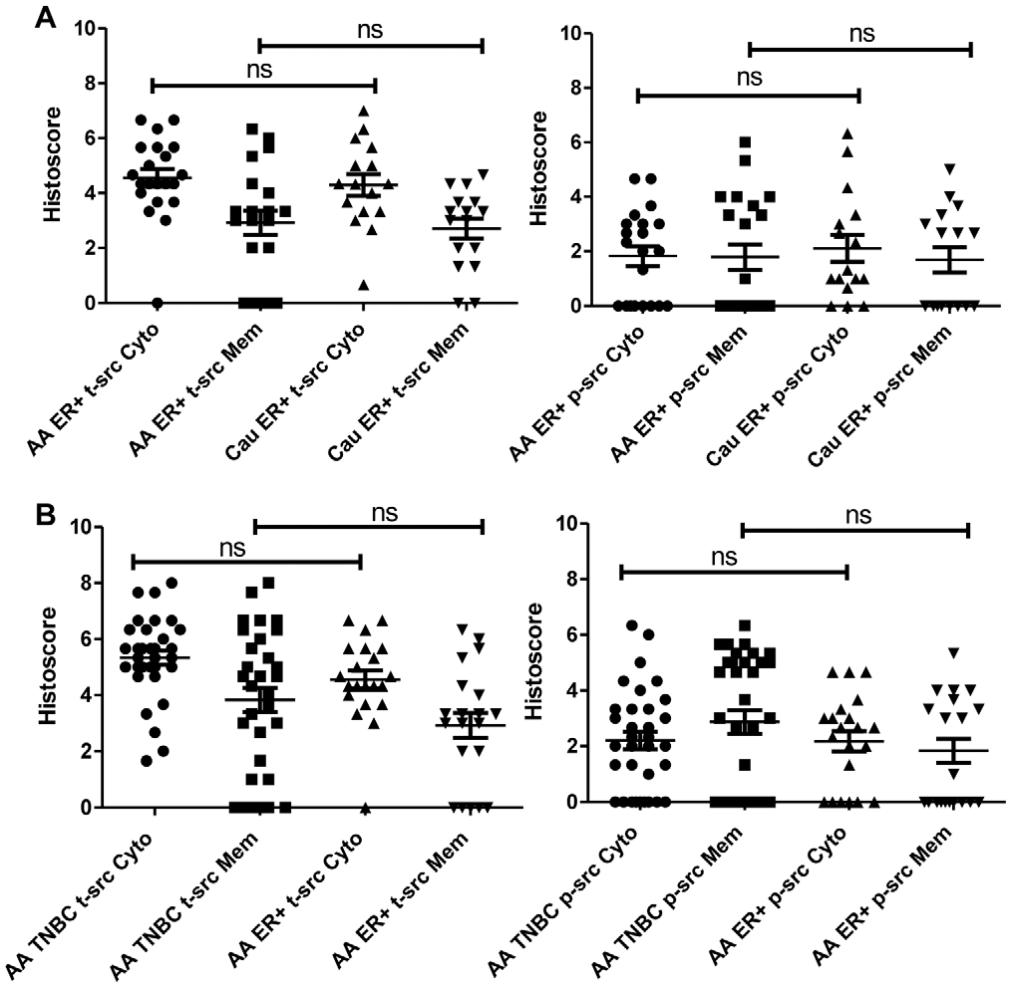
Src expression in African American and Caucasian breast cancer. A) Comparison of total Src (t-src) subcellular localization in the cytoplasm (Cyto) and membrane (Mem) among African American (AA) and Caucasian (Cau) ER+BC cases. B) Comparisons of p-Y416Src (p-Src) localization (cytoplasmic/membrane) among African American (AA) and Caucasians in ER+BC. C) Comparison of total Src subcellular localization (cytoplasmic/membrane) among TNBC and ER+BC in African Americans. D) Comparisons of p-Src subcellular localization (cytoplasmic/membrane) among TNBC and ER+BC in African Americans. Statistical differences in the distribution of total and p-Src kinase were analyzed by the Mann-Whitney *U* test. ns - not significant. The graphs represent the mean histoscore ± SEM (standard error of mean).

There was no significant difference in the expression or localization of either total Src or p-Y416Src in ER+BC between African American (n = 21) and Caucasian (n = 16) cases (Fig. 2A). The comparison of Src expression in TNBC between African American (n = 33) and Caucasian cases (n = 4) was not done due to insufficient Caucasian sample size available from Tulane University Hospital and Clinics.

### Src expression and subcellular localization among African American TNBC and ER+BC

No significant difference was detected in total Src and p-Y416Src expression and subcellular localization between African American TNBC (n = 33) and African American ER+BC (n = 21) cases (Fig. 2B), although African American TNBC exhibited a trend towards an increase in the expression of cytoplasmic total Src (P = 0.060) and membrane p-Y416Src (P = 0.095) as compared to African American ER+BC.

### Src and p-Y416Src expression and subcellular localization in TNBC and ER+BC regardless of race

**Figure 3:**
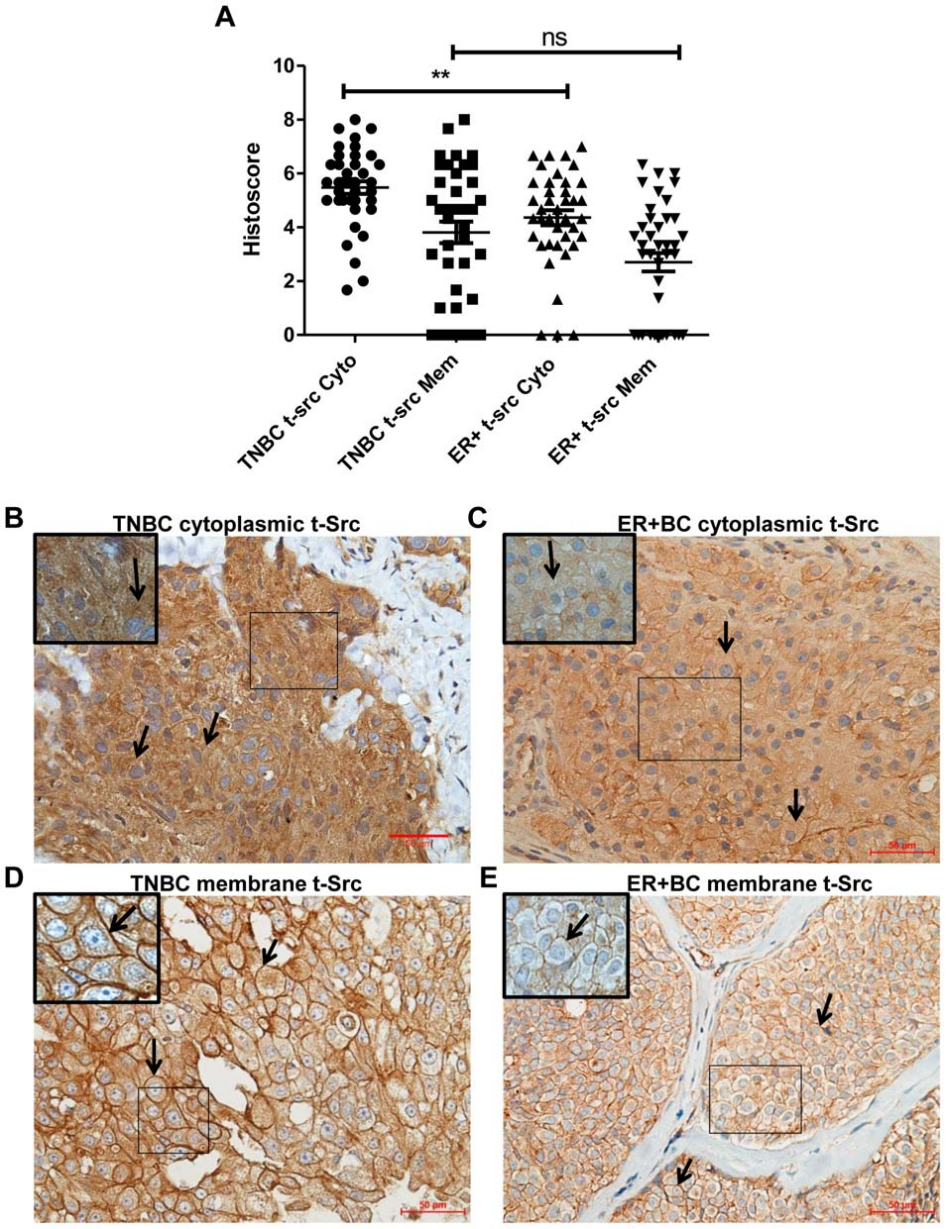
Evaluation of total Src expression in TNBC and ER+BC. A) Comparison of subcellular localization in the cytoplasm (Cyto) and membrane (Mem) of total Src (t-src) in TNBC and ER+BC. B) Representative TNBC case showing strong cytoplasmic (3+) t-Src staining (shown by arrows) (original magnification ×200). C) Representative ER+BC case showing moderate cytoplasmic (2+) t-Src staining (shown by arrows) (original magnification ×200). D) Representative TNBC case showing strong membrane (3+) t-Src staining (shown by arrows) (original magnification ×200). E) Representative ER+BC case showing moderate membrane (2+) t-Src staining (shown by arrows) (original magnification ×200). Statistical differences in the distribution of t-Src in TNBC and ER+BC was analyzed by the Mann-Whitney *U* test, **P<0.01, ns - not significant. *Scale bar*, *50 *µ*m*. Insets are magnified images from selected areas *(small squares)* to show the membrane and cytoplasmic Src staining clearly.

**Figure 4:**
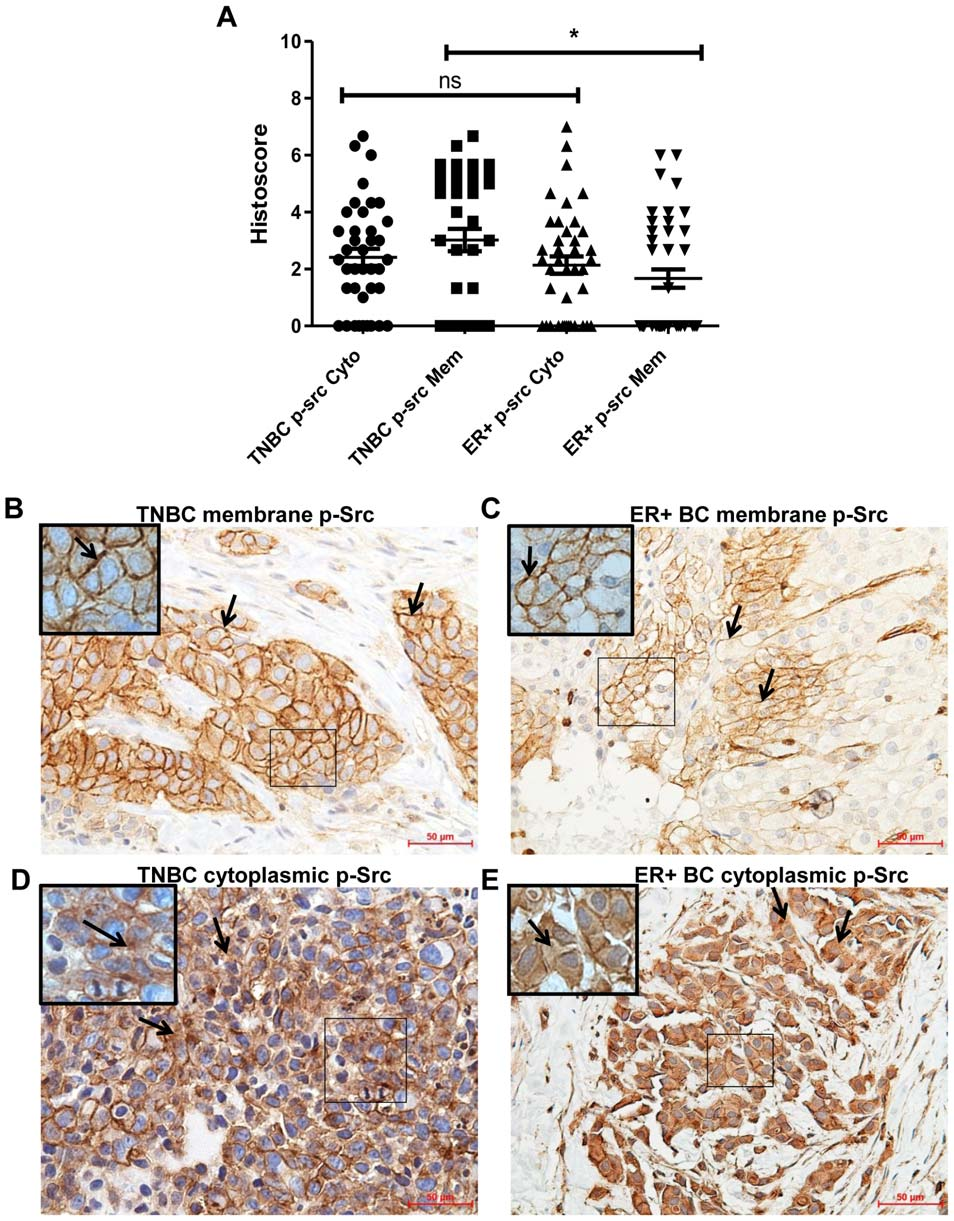
Evaluation of active Src expression in TNBC and ER+BC. A) Comparison between subcellular localization in the cytoplasm (Cyto) and membrane (Mem) of p-Y416Src (p-Src) in TNBC and ER+BC. B) Representative TNBC case showing strong membrane (3+) p-Src staining (shown by arrows) (original magnification ×200). C) Representative ER+BC case showing moderate membrane (2+) p-Src staining (shown by arrows) (original magnification ×200). D) Representative TNBC case showing moderate cytoplasmic (2+) p-Src staining (shown by arrows) (original magnification ×200). E) Representative ER+BC case showing moderate cytoplasmic (2+) staining of p-Src (shown by arrows) (original magnification ×200). Statistical differences in the distribution of p-Src in TNBC and ER+BC was analyzed by the Mann-Whitney *U* test, * P<0.05. ns - not significant. *Scale bar*, *50 *µ*m*. Insets are magnified images from selected areas *(small squares)* to show the membrane and cytoplasmic Src staining clearly.

The comparison of TNBC (n = 39) and ER+BC (n = 40) cases regardless of race revealed that total Src expression in the cytoplasm was significantly higher in TNBC compared to ER+BC (P = 0.0028) (Fig. 3A–C). No significant difference was observed in total Src expression in the membrane for TNBC versus ER+BC (P = 0.2083) (Fig. 3A, D, E). Conversely, p-Y416Src expression in the membrane was significantly higher (P = 0.0106) in TNBC when compared to ER+BC (Fig. 4A–C). No difference was observed in cytoplasmic p-Y416Src between TNBC or ER+BC cases (P = 0.5415) (Fig. 4A, D, E).

### Impact of age, tumor grade, proliferation status and lymph node metastasis on Src kinase expression and subcellular localization

TNBC cases that were positive for lymph node metastasis (n = 13) exhibited higher membrane p-Y416Src compared to lymph node negative TNBC cases (n = 10) (P = 0.027) (Table 3), whereas cytoplasmic p-Y416Src expression in lymph node positive TNBC cases and lymph node negative TNBC cases were not significantly different (P = 0.656) (Table 3). In ER+BC, there was no statistically significant difference in subcellular localization of total Src or p-Y416Src expression between lymph node positive and lymph node negative cases (Table 4). Moreover, total Src and p-Y416Src expression in both ER+BC and TNBC did not exhibit significant differences in subcellular localization when comparing age (women of age≤50 and women of age >50) (Table S1A, B), tumor grade (1, 2 and 3) (Table S2A, B), or proliferation status (high and low Ki67 index) (Table S3A, B). These data indicated that elevated membrane p-Y416Src was detected in TNBC cases with lymph node metastasis, but Src expression/activity and subcellular localization was not associated with age, tumor proliferation or tumor grade in either TNBC or ER+BC in this data set.

**Table 3:** Relationship between lymph node status and Src expression/activity/localization in TNBC.

Variable	Lymph node positive (n = 13)	Lymph node negative (n = 10)	P value
Cytoplasmic total Src	5.74±0.56	5.14±0.29	0.143
Membrane total Src	4.54±0.64	3.03±0.82	0.110
Cytoplasmic p-Y416Src	2.49±0.51	2.23±0.80	0.656
Membrane p-Y416Src	3.23±0.66	0.76±0.58	0.027*

Mean histoscore values ± SEM (standard error of mean) were calculated for total Src and p-Y416Src expression in lymph node positive and negative cases of TNBC. Statistical differences in the distribution of Src and p-Y416Src in TNBC were calculated using the Mann-Whitney *U* test.

* P<0.05 was considered statistically significant.

**Table 4:** Relationship between lymph node status and Src expression/activity/localization in ER+BC.

Variable	Lymph node positive (n = 8)	Lymph node negative (n = 8)	P value
Cytoplasmic total Src	4.66±0.37	2.60±0.91	0.220
Membrane total Src	2.88±0.72	1.89±0.90	0.410
Cytoplasmic p-Y416Src	1.60±0.47	1.67±0.86	0.822
Membrane p-Y416Src	1.12±0.71	1.08±0.73	0.945

Mean histoscore values ± SEM (standard error of mean) were calculated for total Src and p-Y416Src expression in lymph node positive and negative cases of ER+BC. Statistical differences in the distribution of Src and p-Y416Src in ER+BC were calculated using the Mann-Whitney *U* test. *P<0.05 was considered statistically significant.

## Discussion

Although a number of studies have examined Src kinase expression in breast cancer [24], [25], [26], [27], to our knowledge, no studies have combined investigation of Src expression, activity and localization in comparing TNBC with ER+BC, cancer tissue with normal epithelial ducts, and Caucasian with African American ethnic groups. In addition to measuring total Src expression, it is important to measure the level of active Src [27] and evaluate membrane localization, since these latter two assessments may be more appropriate biomarkers for predicting clinical response to Src kinase inhibitors. Currently, p-Y416Src is most commonly assessed to determine the level of active Src. The present single site study using samples from breast cancer patients seen at the Tulane University Hospital and Clinics, New Orleans, Louisiana, found that both cytoplasmic and membrane Src were detected in the majority of breast cancer cases. The systematic examination of expression, activity and subcellular localization of Src in TNBC and ER+BC demonstrated that total Src expression was higher in cancer when compared to adjacent normal ducts in both the cell membrane and the cytoplasm. Active p-Y416Src was elevated in the membranes of cancer cells compared to normal ducts. Total Src in the cytoplasm and p-Y416Src in the membrane was elevated in TNBC compared to ER+BC. No difference in the expression and activity of Src at any cellular location was found when age (≤50 versus>50), high/low ki67 index, or tumor grade (1, 2 and 3) was compared. TNBC cases with positive lymph node metastasis showed elevated p-Y416Src in the membranes of cancer cells compared to TNBC cases that were negative for lymph node metastasis. These data demonstrate that elevated Src expression correlated with more aggressive TNBC, although no racial/ethnic differences in Src were observed within the limited data set of ER+BC.

Subcellular localization of Src is a key regulatory mechanism for control of Src activation. Upon external stimuli, inactive Src in the cytoplasm is translocated to the cell membrane and activated by phosphorylation [14], [15], [16], [17]. The p-Y416Src at the cell membrane has been shown to initiate intracellular signal transduction pathways that influence cell adhesion and initiate cell migration [18]. A study by Reissig et. al. that found elevated Src expression and activity in cancer tissue when compared with adjacent matched normal tissue in 25 of 52 breast cancer cases but did not determine the subcellular localization of Src and active Src [25]. The present study provides additional details regarding the subcellular localization of total and active Src. We report that p-Y416Src expression in the membrane was significantly higher in TNBC compared to ER+BC. A previous study by Tryfonopoulos et al. reported an increase of total Src in membrane of TNBC compared to non-TNBC cases, but did not address active Src [28]. In addition to elevated active Src in the membrane of TNBC cells, there was a higher level of total Src in the cytoplasm of TNBC compared to ER+BC. Studies have shown that breast cancer patients with elevated total Src in the cytoplasm had a lower disease specific survival compared to patients with low cytoplasmic Src [29]. Several studies have demonstrated that TNBC has a poor survival outcome compared to ER+BC [3], [4]. The elevated detection of p-Y416Src in the membrane of TNBC coupled with elevated total Src in the cytoplasm of TNBC is consistent with the more aggressive and metastatic phenotype of TNBC compared to other breast cancer subtypes [22].

In the present study neither TNBC nor ER+BC showed any significant differences in expression, activity or subcellular localization of Src when comparing age, tumor grade or Ki67 status. A higher level of p-Y416Src (active form) was detected in the membrane of cancer cells from TNBC patients that had lymph node positive disease as compared to patients classified as lymph node negative. This is consistent with the known role of c-Src in cancer cell invasion and metastasis. In addition, p-Y416Src was localized to the membrane more frequently in more aggressive TNBC specimens as compared to the less aggressive ER+BC specimens. These data suggested that membrane localized active Src may be correlated with aggressive and/or metastatic disease and are consistent with previous studies comparing Src expression in other breast cancer subtypes. A recent study demonstrated that high expression of p-Y416Src was associated with breast cancer metastatic disease although cytoplasmic/membrane localization of activated p-Y416Src was not assessed [30]. Other studies have shown that cancer cell lines with elevated Src activity are frequently highly metastatic, exhibiting an elevated migration and invasion potential *in vitro* [31], [32], [33]. Taken together, the present study found that p-Y416Src (active form) was localized to the membrane more frequently in aggressive TNBC as compared to less aggressive ER+BC.

With the understanding that Src may promote breast cancer invasiveness, it might be expected that clinical Src inhibitors could be effective in inhibiting metastatic breast cancer. However to date, the response rates with clinical Src inhibitors in unselected breast cancer patient groups have been very modest [34], [35], [36]. A phase II clinical trial using the broad specificity Src kinase inhibitor dasatinib as a single agent in patients with metastatic TNBC showed only a modest response rate of 5% [34], [35]. A phase II trial using another broad specificity Src inhibitor saracatinib in hormone receptor negative breast cancer patients (n = 9) did not provide positive results; no patient achieved complete response (CR), partial response (PR), or stable disease (SD) at >6 months [36]. It is unknown why these clinical Src inhibitors did not result in a more significant response rate. It is noted that Src expression in tissue biopsies from patients in these studies was not assessed. The Src inhibitors used in these studies represent the ATP analogue class of inhibitors that bind in the ATP-binding pocket of Src and many other tyrosine kinases and hence do not exhibit complete specificity for Src kinase. It is unknown whether the broad specificity of current ATP analogue Src inhibitors may underlie problems with efficacy in breast cancer patients. These studies indicate a need for stratification of patients to identify those who are likely to respond to a Src inhibitor. More understanding about the level and activity of Src related to subcellular localization, race/ethnicity, and relationship to breast tumor subtypes may permit clinicians to better predict patients who will respond to Src kinase inhibitors.

This study compared all TNBC to ER+BC cases and was not restricted to any pathological subtype. Three TNBC cases were DCIS and five ER+BC cases were DCIS. These DCIS cases were useful in evaluating Src expression in the progression from normal breast epithelium, to DCIS and invasive disease. Recently, Humar et. al. detected increased levels of active Src (p-Y416Src) in invasive lobular carcinoma (LBC) relative to non-invasive lobular carcinoma *in situ* (LCIS) or non-neoplastic epithelia in LBC patients [37] suggesting that the acquisition of invasiveness coincides with increased active Src in the membrane. In the present study only four LBC cases were identified that were also TNBC; active Src was not detected in the membrane of these tumors although no conclusions can be made due to low sample number. The majority of the cases in the present study were invasive ductal carcinoma that showed elevated levels of Src and active Src (p-Y416Src) in cancer cell membrane relative to adjacent normal ducts. Of the eight cases that had regions of ductal carcinoma *in situ* (DCIS), five of these DCIS cases showed increased active Src in the membrane of the DCIS regions. The present data evaluating ductal carcinomas are consistent with the data on lobular carcinoma in which Src and active Src are increased with the progression from benign, to *in situ*, to invasive disease. Src protein expression was not detected in the cell nucleus in the present study or in two other previous studies [28], [30]. However, Elsberger et. al. detected total Src nuclear staining in 48% and p-SrcY416 in 92% of breast cancer cases [27]. Another study by the same group detected active Src within the nucleus of tumor cells in over 50% of breast cancer cases that was significantly associated with improved patient survival and decreased recurrence in tamoxifen-treated patients with ER+BC [26]. A limitation of the present study was lack of long-term follow up data for the breast cancer cases. All cases were from recent years (2007–2009) and long-term follow-up on recurrence and data to calculate survival curves were not available. Older archival specimens were not available from the tumor bank because of sustained flooding that occurred in late 2005 from Hurricane Katrina.

In ER+BC, a comparison between African American and Caucasian cases revealed no significant differences in the expression, activity and localization of Src between these ethnic groups. Breast cancer patients seen in the Tulane University Hospital and Clinics are predominantly African American women who present with TNBC with few Caucasian women who present with TNBC. The number of available cases of Caucasian women who presented with TNBC (n = 4) 2007–2009 was not a sufficient sample size to make conclusions about racial/ethnic differences in Src expression in TNBC. Within all the African American breast cancer cases, total and active Src was elevated in TNBC compared to ER+BC.

## Materials and Methods

### Ethics statement

The specimens to be used in this research project were obtained under IRB protocol #10-169449 approved by the Tulane University Institutional Review Board (IRB). No patient identifiers were associated with the samples used in this study. This study used archival biopsies of breast cancer and there was no recruitment of patients. Specimens were used from women who have previously consented to donate biological specimens to the Tulane Hospital specimen repository.

### Tissue cases

A total of 79 paraffin tissue samples were obtained from patients who were diagnosed with breast cancer at Tulane University Hospital and Clinics between 2007 and 2009. All the breast cancer cases were procured from the Department of Pathology, Tulane Health Sciences Center with IRB approval. Since all breast cancer cases were from recent years, long-term follow-up data to calculate survival curves was not available. In the present study all tumor samples or biopsies from breast cancer patients were collected and transferred to 10% neutral buffered formalin within 15 minutes of excision to minimize loss of phospho-antigens. Race, age, tumor type, size, grade, Ki67 status, lymph node status, and ERα/PR/HER2/neu expression were obtained from the pathology case reports. Thirty-nine (39) cases represented TNBC (33 African Americans, 4 Caucasians, 2 race unknowns) and 40 cases represented ER+BC (21 African Americans and 16 Caucasians, 3 race unknowns).

#### TNBC cases

All TNBC cases were negative for ERα/PR expression, and Her2/neu amplification/overexpression. Tumor type: 33 cases were invasive ductal carcinoma, 3 cases were ductal carcinoma in situ (DCIS), 1 case was adenoid cystic carcinoma, 1 case anaplastic giant cell carcinoma and 1 case was mucinous carcinoma. Tumor grade: 1 case was grade 1, 7 cases were grade 2, 28 cases were grade 3. Ki67 expression: 31 cases had a high Ki67 index (>20% Ki67 staining), 4 cases had a low Ki67 index (≤20% staining), 4 unknown. Lymph node status: 13 cases were lymph node positive and 10 cases were lymph node negative; for the remaining 24 cases lymph node status was not checked. Age: Of 39 TNBC cases, 20 cases were over 50 years of age, 19 cases were under 50 years of age.

#### ER+BC cases

All cases were PR positive and ERα status was 90% positive or greater. 16 cases had PR status of 90% and 24 cases had less than 90% PR staining. Tumor type: 29 cases were invasive ductal carcinoma, 4 cases were invasive lobular carcinoma, 5 cases were DCIS, 1 case was papillary carcinoma, and 1 case was mucinous adenocarcinoma. Tumor grade: 9 cases were grade 1, 19 cases were grade 2, and 7 cases were grade 3. Ki67 status: 25 cases had a low Ki67 index (≤20% staining), 13 cases had a high Ki67 index (>20% Ki67 staining), and 2 cases were unknown. Lymph node status: 8 cases were lymph node positive and 8 cases were lymph node negative, for the remaining 24 cases lymph node status was not determined. Age: Of 40 ER+BC cases, 29 cases were over 50 years of age, 11 cases were under 50 years of age.

### Immunohistochemistry

Total Src kinase and activated phospho Src kinase expression (p-Y416Src) was assessed by IHC using antibodies against total c-Src (36D10; CAT # 2109, Cell Signaling Technology, Beverly, MA) and p-Y416Src (CAT # 2101, Cell Signaling Technology, Beverly, MA). These antibodies have been used in several studies that measured Src expression and activity by IHC [30], [38]. Immunohistochemistry (IHC) was performed as previously described in our laboratory [39]. Briefly after deparaffinization and rehydration of sections, antigen retrieval was performed by immersing slides in Borg Decloaker (antigen retrieval solution) (Biocare Medical, Concord, CA) and heating in a steam cooker for 20 minutes followed by cooling for 20 minutes at room temperature. Endogenous peroxidase activity in tissue sections was inhibited by incubation in 3% hydrogen peroxide (H_2_O_2_) for 5 minutes. To reduce nonspecific binding, tissue sections were subsequently incubated with background sniper (Biocare Medical, Concord, CA) for 5 minutes at room temperature. Slides were then incubated overnight at 4°C with antibody to c-Src (1∶200 dilution in PBS) or phospho-Y419Src (1∶100 dilution in PBS). Signal was amplified and visualized using MACH 4 Universal HRP Polymer Detection (Biocare Medical, Concord, CA) and the chromogen 3, 3′-diaminobenzidine (DAB) (Vector Laboratories Burlingame, CA). Sections were counterstained with hematoxylin, dehydrated, and mounted. To evaluate the specificity of immunostaining, the primary Src antibody or phospho-Y419Src antibody was replaced with non-specific, isotyped matched rabbit IgG diluted in PBS. Specificity of all antibodies was confirmed by Western blotting.

### Scoring

Src expression in tumor cases was measured by IHC and assessed using the histoscore method developed by Allred et al. [41]. Staining for Src in each cellular location (cytoplasm and membranes) was scored separately for each sample. For each specimen, a proportion score and an intensity score were determined. The intensity of immunostaining was scored based on a visual assessment of the staining intensity (brown color) within the cytoplasm or cell membrane on a scale of 0 (no staining) to 3+ (intense staining). The intensity score represented the staining intensity in positively stained cells (0 = none; 1 = + weak; 2 = ++ intermediate, 3 = +++ strong). The proportion score represented the percentage of positively stained cells by microscopic examination of the entire tissue section (0 = none; 1 = <5%; 2 = 5–25%; 3 = 26–50% 4 = 51–75% 5 = >75%) [40]. For example, when only cytoplasmic, but not membrane Src staining was detected uniformly throughout a tissue section, the sample was given a proportion score of 5 for cytoplasm and a proportion score of 0 for membrane. Similarly when only membrane, but not cytoplasmic Src staining was detected uniformly throughout a tissue section, the sample was given a proportion score of 0 for cytoplasm and a proportion score of 5 for membrane. If both cytoplasm and membrane were stained at approximately equivalent proportions (50%) in a tissue section, a proportion score of 3 was given to both cytoplasm and membrane. The overall Src kinase expression in each tumor sample was expressed as a histoscore that was the sum of the proportion score (0–5) and the intensity score (0–3) for a range of between 0–8, with a maximum possible score of 8 [41].Three different observers M.A., A.A. and B.G.R. were trained by pathologist KM to score the slides independently and agreement between the observers was consistent.

### Statistical analysis

Values were reported as mean± SE. The Mann-Whitney *U* test was used to analyze the differences in the expression of active and total Src in cytoplasm and membrane between normal ducts and cancer tissue, between African American and Caucasian cases, between low and high proliferative index (Ki67 index), and between positive and negative lymph node status. The Kruskal-Wallis H test was used to compare the expression of active and total Src between grades of carcinoma. The Chi-square test was used to estimate frequency of expression, activity and subcellular localization of Src between TNBC and ER+BC. Statistical analyses were performed using Graph pad Prism 5. A P value less than 0.05 was considered statistically significant.

## Supporting Information

Table S1 Expression of Src kinase in different age groups of TNBC and ER+BC. Mean histoscore values ± SEM (standard error of mean) were calculated for total Src and p-Y416Src expression in patients ≤50 or >50 years of age for A) TNBC and B) ER+BC. Statistical differences in the distribution of Src and p-Y416Src in TNBC and ER+BC were calculated using the Mann-Whitney *U* test. *P<0.05 was considered statistically significant.
(DOC)

Table S2 Expression of Src kinase related to tumor grade in TNBC and ER+BC. Mean histoscore values ± SEM (standard error of mean) were calculated for total Src and p-Y416Src expression related to tumor grade for A) TNBC, and B) ER+BC. Statistical differences in the distribution of Src and p-Y416Src in grades 2 and 3 TNBC were calculated using the Mann-Whitney *U* test. Statistical differences in the distribution of Src and p-Y416Src in grades 1, 2 and 3 of ER+BC were calculated by the Kruskal-Wallis H test. *P<0.05 was considered statistically significant.
(DOC)

Table S3 Expression of Src kinase related to low and high Ki67 status in TNBC and ER+BC. Mean histoscore values ± SEM (standard error of mean) were calculated for total Src and p-Y416Src expression in patients with low or high Ki67 status for A) TNBC and B) ER+BC. Statistical differences in the distribution of Src and p-Y416Src in TNBC and ER+BC were calculated using the Mann-Whitney *U* test. *P<0.05 was considered statistically significant.
(DOC)
