# Supplementary material for: Subcellular Localization of Total and Activated Src Kinase in African American and Caucasian Breast Cancer
Source: PLoS One. 2012 Mar 22;7(3):e33017. doi: 10.1371/journal.pone.0033017 (PMC3310861; doi:10.1371/journal.pone.0033017)
Supplement: Table S1 — Expression of Src kinase in different age groups of TNBC and ER+BC. Mean histoscore values ± SEM (standard error of mean) were calculated for total Src and p-Y416Src expression in patients ≤50 or >50 years of age for A) TNBC and B) ER+BC. Statistical differences in the distribution of Src and p-Y416Src in TNBC and ER+BC were calculated using the Mann-Whitney U test. *P<0.05 was considered statistically significant. (DOC) [file pone.0033017.s001.doc]

**Table S1** A) Relationship between different age group and Src expression in TNBC

| **Variable** | **Age ≤50 (n = 19)** | **Age >50 (n = 20)** | **P value** |
| --- | --- | --- | --- |
| Total Src cytoplasm | 5.59 ± 0.37 | 5.14 ± 0.47 | 0.544 |
| Total Src membrane | 3.28± 0.65 | 4.15± 0.56 | 0.364 |
| p-Y416Src cytoplasm | 2.88 ± 0.48 | 3.04 ± 0.52 | 0.733 |
| p-Y416Src membrane | 2.55± 0.56 | 3.07 ± 0.59 | 0.496 |

B) Relationship between different age group and Src expression in ER+BC

| **Variable** | **Age ≤50 (n = 11)** | **Age >50 (n = 29)** | **P value** |
| --- | --- | --- | --- |
| Total Src cytoplasm | 4.46 ± 0.72 | 4.06 ± 0.36 | 0.308 |
| Total Src membrane | 1.88 ± 0.62 | 2.67 ± 0.43 | 0.451 |
| p-Y416Src cytoplasm | 2.05 ± 0.59 | 2.08 ± 0.40 | 0.988 |
| p-Y416Src membrane | 1.63 ± 0.72 | 1.52 ± 0.37 | 0.986 |

**Table S1:** Expression of Src kinase in different age groups of TNBC and ER+BC. Mean histoscore values ± SEM (standard error of mean) were calculated for total Src and p-Y416Src expression in patients ≤50 or >50 years of age for A) TNBC and B) ER+BC. Statistical differences in the distribution of Src and p-Y416Src in TNBC and ER+BC were calculated using the Mann-Whitney *U* test. *P< 0.05 was considered statistically significant.
