# Supplementary material for: Subcellular Localization of Total and Activated Src Kinase in African American and Caucasian Breast Cancer
Source: PLoS One. 2012 Mar 22;7(3):e33017. doi: 10.1371/journal.pone.0033017 (PMC3310861; doi:10.1371/journal.pone.0033017)
Supplement: Table S2 — Expression of Src kinase related to tumor grade in TNBC and ER+BC. Mean histoscore values ± SEM (standard error of mean) were calculated for total Src and p-Y416Src expression related to tumor grade for A) TNBC, and B) ER+BC. Statistical differences in the distribution of Src and p-Y416Src in grades 2 and 3 TNBC were calculated using the Mann-Whitney U test. Statistical differences in the distribution of Src and p-Y416Src in grades 1, 2 and 3 of ER+BC were calculated by the Kruskal-Wallis H test. *P<0.05 was considered statistically significant. (DOC) [file pone.0033017.s002.doc]

**Table S2**A) Relationship between histological grade and Src expression/activity/localization in TNBC

| **Variable** | **Grade 2 (n = 7)** | **Grade 3 (n = 28)** | **P value** |
| --- | --- | --- | --- |
| Total Src cytoplasm | 5.48 ± 0.97 | 5.66 ± 0.21 | 0.419 |
| Total Src membrane | 3.91 ± 1.13 | 3.93 ± 0.45 | 0.885 |
| p-Y416Src cytoplasm | 3.43 ± 0.74 | 3.09 ± 0.42 | 0.934 |
| p-Y416Src membrane | 3.38 ± 0.86 | 2.86 ± 0.48 | 0.555 |

B) Relationship between histological grade and Src expression/activity/localization in ER+BC

| **Variable** | **Grade 1 (n= 9)** | **Grade 2 (n= 19)** | **Grade 3 (n = 7)** | **P value** |
| --- | --- | --- | --- | --- |
| Total Src cytoplasm | 5.09 ± 0.32 | 4.25 ± 0.50 | 3.93 ± 0.77 | 0.489 |
| Total Src membrane | 3.05 ± 0.74 | 2.37 ± 0.52 | 2.01 ± 0.74 | 0.587 |
| p-Y416Src cytoplasm | 2.26 ± 0.76 | 1.86 ± 0.49 | 2.38 ± 0.53 | 0.764 |
| p-Y416Src membrane | 0.82 ± 0.66 | 1.43 ± 0.46 | 2.24 ± 0.88 | 0.396 |

**Table S2:** Expression of Src kinase related to tumor grade in TNBC and ER+BC. Mean histoscore values ± SEM (standard error of mean) were calculated for total Src and p-Y416Src expression related to tumor grade for A) TNBC, and B) ER+BC. Statistical differences in the distribution of Src and p-Y416Src in grades 2 and 3 TNBC were calculated using the Mann-Whitney *U* test. Statistical differences in the distribution of Src and p-Y416Src in grades 1, 2 and 3 of ER+BC were calculated by the Kruskal-Wallis H test. *P < 0.05 was considered statistically significant.
