# Supplementary material for: Subcellular Localization of Total and Activated Src Kinase in African American and Caucasian Breast Cancer
Source: PLoS One. 2012 Mar 22;7(3):e33017. doi: 10.1371/journal.pone.0033017 (PMC3310861; doi:10.1371/journal.pone.0033017)
Supplement: Table S3 — Expression of Src kinase related to low and high Ki67 status in TNBC and ER+BC. Mean histoscore values ± SEM (standard error of mean) were calculated for total Src and p-Y416Src expression in patients with low or high Ki67 status for A) TNBC and B) ER+BC. Statistical differences in the distribution of Src and p-Y416Src in TNBC and ER+BC were calculated using the Mann-Whitney U test. *P<0.05 was considered statistically significant. (DOC) [file pone.0033017.s003.doc]

**Table S3** A) Relationship between Ki67 status and Src expression/activity/localization in TNBC

| **Variable** | **Ki67** **Low** (**n = 4)** | **Ki67** **High** (**n = 31)** | **P value** |
| --- | --- | --- | --- |
| Total Src cytoplasm | 5.92 ± 0.49 | 5.46 ± 0.30 | 0.814 |
| Total Src membrane | 4.67 ± 1.46 | 3.97 ± 0.45 | 0.496 |
| p-Y416Src cytoplasm | 3.76 ± 1.37 | 3.14 ± 0.37 | 0.714 |
| p-Y416Src membrane | 3.00 ± 1.50 | 3.17 ± 0.42 | 0.731 |

B) Relationship between Ki67 status and Src expression/activity/localization in ER+BC

| **Variable** | **Ki67** **Low (n = 25)** | **Ki67** **High (n = 13)** | **P value** |
| --- | --- | --- | --- |
| Total Src cytoplasm | 4.07 ± 0.42 | 4.17 ± 0.56 | 0.840 |
| Total Src membrane | 2.16 ± 0.43 | 2.70 ± 0.65 | 0.597 |
| p-Y416Src cytoplasm | 2.07 ± 0.37 | 2.01 ± 0.61 | 0.849 |
| p-Y416Src membrane | 1.35 ± 0.35 | 1.62 ± 0.60 | 0.861 |

**Table S3:** Expression of Src kinase related to low and high Ki67 status in TNBC and ER+BC. Mean histoscore values ± SEM (standard error of mean) were calculated for total Src and p-Y416Src expression in patients with low or high Ki67 status for A) TNBC and B) ER+BC. Statistical differences in the distribution of Src and p-Y416Src in TNBC and ER+BC were calculated using the Mann-Whitney *U* test. *P< 0.05 was considered statistically significant.
